# Rescue technique after endoscopic ultrasound-guided hepaticogastrostomy stent dislocation

**DOI:** 10.1055/a-2213-1149

**Published:** 2023-12-11

**Authors:** Takeshi Ogura, Taro Iwatsubo, Atsushi Okuda, Saori Ueno, Hiroki Nishikawa

**Affiliations:** 1 38588 Endoscopy Center, Osaka Medical and Pharmaceutical University Hospital, Takatsuki, Japan; 2 13010 2nd Department of Internal Medicine, Osaka Medical and Pharmaceutical University, Takatsuki, Japan

Endoscopic ultrasound-guided hepaticogastrostomy (EUS-HGS) has been indicated after failed endoscopic retrograde cholangiopancreatography (ERCP). Critical adverse events such as stent migration or dislocation can occur during EUS-HGS 1 2 3 . If a fistula is not formed after the occurrence of these adverse events, an alternative biliary drainage technique is required, such as percutaneous transhepatic biliary drainage and endoscopic suture of the anastomotic defect left behind after the EUS-HGS stent. A novel endoscopic suture using an endoloop and endoclips has recently been reported for use after upper gastrointestinal perforations 4 5 . We herein describe the successful use of these techniques for endoscopic treatment after EUS-HGS stent dislocation.

A 57-year-old man was admitted with obstructive jaundice due to inoperable pancreatic cancer. Because the second part of the duodenum was obstructed, EUS-HGS was performed using a partially covered self-expandable metal stent. However, abdominal pain and recurrence of obstructive jaundice were noted 5 days later, and CT revealed dislocation of the stent ( Fig. 1 ). After insertion of the duodenoscope in endoscopic re-intervention, attempts to insert the guidewire into the intrahepatic bile duct were unsuccessful ( Fig. 2 ). Therefore, EUS-guided choledochoduodenostomy was performed. The EUS-HGS stent was then removed. A single-channel endoscope was inserted to perform endoscopic suturing. After locating the anastomotic defect left behind after the EUS-HGS stent ( Fig. 3 ), an endoloop was grasped by the tip of the endoscope from the outside and advanced to this site along with the endoscope. The endoloop was placed in the open state along the edge of the defect and anchored at multiple sites by clips that allow repeated opening and closing ( Fig. 4 ). Finally, the anastomotic defect was sutured by tightening the fixed endoloop ( Fig. 5 **;** Video 1 ). These procedures were successfully performed without any adverse events.

**Fig. 1:**
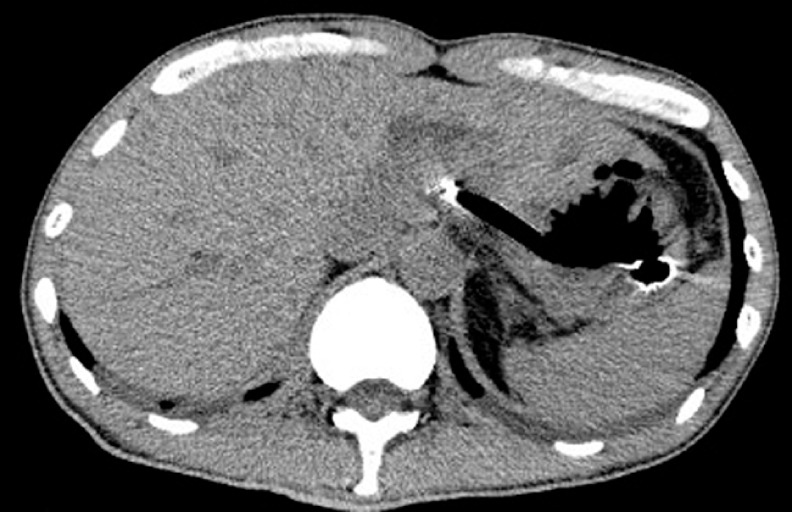
Computed tomography image shows dislocation of an endoscopic ultrasound-guided hepaticogastrostomy (EUS-HGS) stent.

**Fig. 2:**
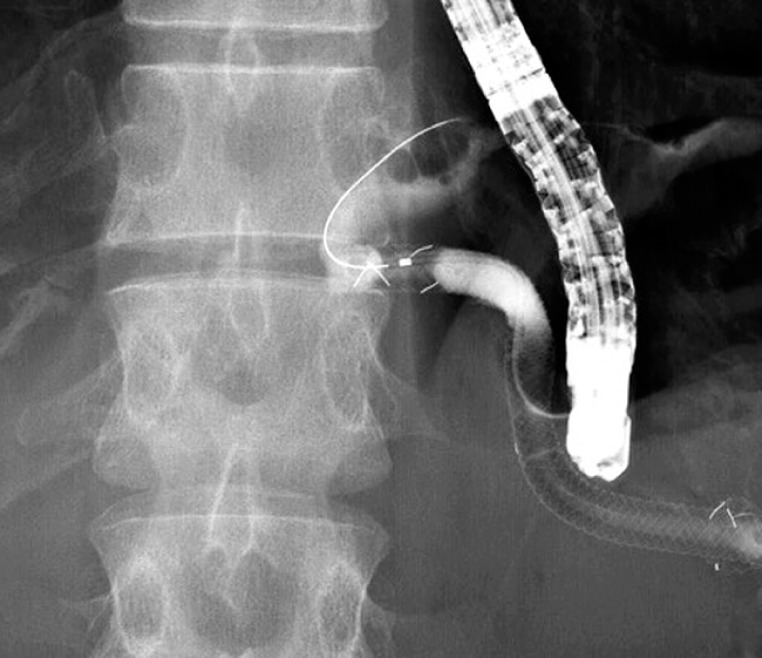
The EUS-HGS stent has become dislocated from the intrahepatic bile duct and leakage of contrast medium into the abdominal cavity is observed.

**Fig. 3:**
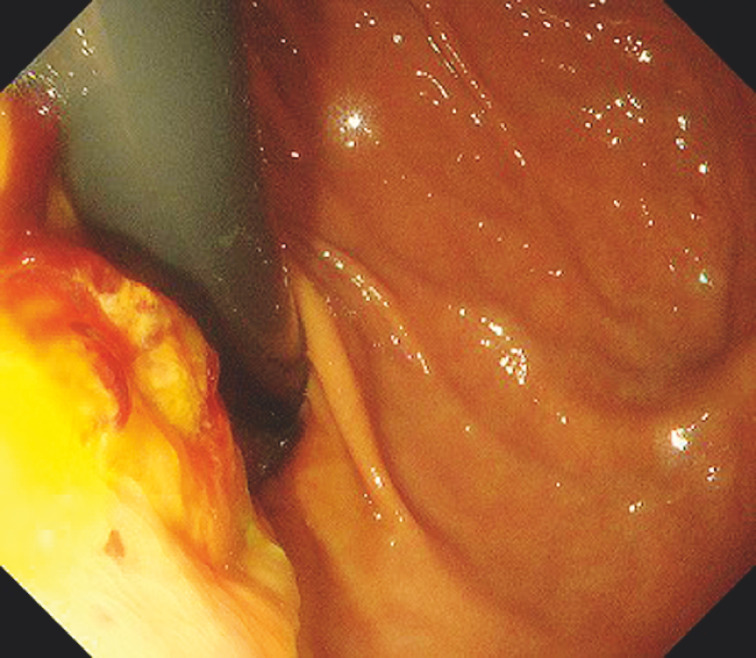
The anastomotic defect is visible after removal of the stent.

**Fig. 4:**
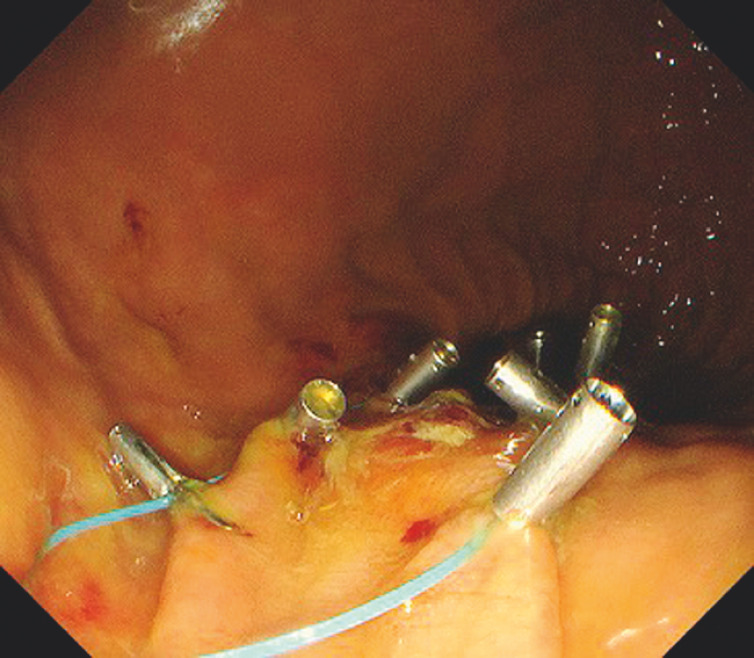
An endoloop is placed in the open state along the edge of the defect and anchored at multiple sites by clips that allow repeated opening and closing.

**Fig. 5:**
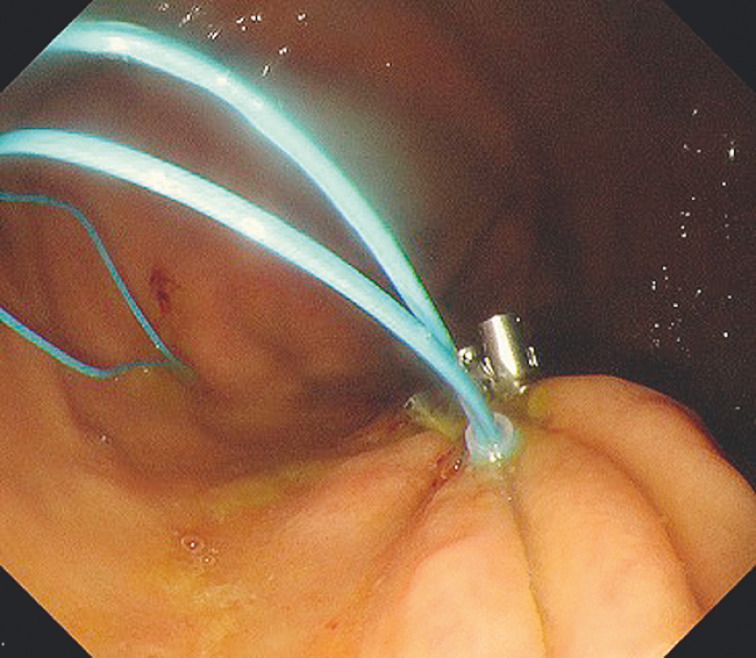
Successful suturing of the anastomotic defect.

Novel endoscopic suture using an endoloop and endoclips successfully deployed for endoscopic treatment after dislocation of an endoscopic ultrasound-guided hepaticogastrostomy stent.Video 1

In conclusion, the present technique may be useful for troubleshooting during EUS-HGS, particularly in the case of stent dislocation.

Endoscopy_UCTN_Code_CPL_1AL_2AD

**Correction: Rescue technique after endoscopic ultrasound-guided hepaticogastrostomy stent dislocation**

**Takeshi Ogura, Taro Iwatsubo, Atsushi Okuda et al. Rescue technique after endoscopic ultrasound-guided hepaticogastrostomy stent dislocation.** Endoscopy 2023, 55: E1236–E1237, doi:10.1055/a-2213-1149

In the above-mentioned article the pagination of the PDF has been corrected. This was corrected in the online version on 22 March, 2024.

## Notes

Endoscopy E-Videos https://eref.thieme.de/e-videos
*E-Videos* is an open access online section of the journal *Endoscopy* , reporting on interesting cases and new techniques in gastroenterological endoscopy. All papers include a high-quality video and are published with a Creative Commons CC-BY license. Endoscopy E-Videos qualify for HINARI discounts and waivers and eligibility is automatically checked during the submission process. We grant 100% waivers to articles whose corresponding authors are based in Group A countries and 50% waivers to those who are based in Group B countries as classified by Research4Life (see: https://www.research4life.org/access/eligibility/ ). This section has its own submission website at https://mc.manuscriptcentral.com/e-videos .

## Notes

Correction: Rescue technique after endoscopic ultrasound-guided hepaticogastrostomy stent dislocation **Takeshi Ogura, Taro Iwatsubo, Atsushi Okuda et al. Rescue technique after endoscopic ultrasound-guided hepaticogastrostomy stent dislocation.** Endoscopy 2023, 55: E1236–E1237, doi:10.1055/a-2213-1149
In the above-mentioned article the pagination of the PDF has been corrected. This was corrected in the online version on 22 March, 2024.
