# Correction: Rescue technique after endoscopic ultrasound-guided hepaticogastrostomy stent dislocation

**DOI:** 10.1055/a-2292-2013

**Published:** 2024-03-26

**Authors:** Takeshi Ogura, Taro Iwatsubo, Atsushi Okuda, Saori Ueno, Hiroki Nishikawa

**Affiliations:** 1 38588 Endoscopy Center, Osaka Medical and Pharmaceutical University Hospital, Takatsuki, Japan; 2 13010 2nd Department of Internal Medicine, Osaka Medical and Pharmaceutical University, Takatsuki, Japan



In the above-mentioned article the pagination of the PDF has been corrected. This was corrected in the online version on 22 March, 2024.

